# Fetal origins of autism spectrum disorders: the non-associated maternal factors

**DOI:** 10.4155/fsoa-2015-0001

**Published:** 2016-03-21

**Authors:** Hind N Moussa, Anand Srikrishnan, Sean C Blackwell, Pramod Dash, Baha M Sibai

**Affiliations:** 1 Department of Obstetrics & Gynecology at The McGovern Medical School at The University of Texas Health Science Center at Houston, Houston, TX, USA; 2 Neurobiology & Anatomy, McGovern Medical School at The University of Texas Health Science Center at Houston, Houston, TX, USA

**Keywords:** autism spectrum disorder, fetal origins, nonassociated maternal factors, population studies

## Abstract

**Aim:**

Several population-based studies have been conducted to determine whether maternal exposures are involved in the pathophysiology of autism spectrum disorder (ASD). We review these studies and describe the factors not associated with increased risk for ASD development.

**Methods:**

We identified studies describing associations between maternal exposures and ASD development. These studies include the Childhood Autism Risks from Genetics and the Environment, Nurses’ Health Study II, and the Swedish population registry.

**Results:**

Factors not associated with ASD development include Type 2 and gestational diabetes, chronic hypertension, fever treated with antipyretic medication, autoimmune disease and short interpregnancy intervals.

**Conclusion:**

There is increasing evidence that maternal exposures are involved in the pathophysiology of ASD in the developing fetus.

## Abstract (lay abstract)

**Lay Abstract:** Autism spectrum disorder currently affects one in 68 children in the USA, with up to one in 42 boys. Its underlying etiology is largely unknown. Although autism was once considered a childhood disease, efforts for early detection led to the realization that it might originate at a much earlier stage, namely fetal life. Several maternal risk factors have recently been studied to establish associations with autism in the offspring. In this review, we present the maternal risk factors that were not found to have such associations.

Autism spectrum disorder (ASD) is defined in the fifth edition of the Diagnostic and Statistical Manual of Mental Disorders (DSM-5) as persistent deficits in social communication and social interaction across multiple contexts, as manifested by deficits in social-emotional reciprocity, deficits in nonverbal communicative behaviors used for social interaction, or deficits in developing, maintaining and understanding relationships [1]. The current ASD prevalence in children is estimated to be approximately 1% [2] but has been reported to be as high as 2.6% [3]. The US Department of Developmental Services reported a 556% increase in the prevalence of autism from 1991 to 1997 [4], a rate that is higher than the prevalence rates reported for other pediatric disorders such as spina bifida, cancer and Down syndrome. The US Environmental Protection Agency (EPA) determined that there was a changepoint at 1987 [5] for increasing autistic disorder (AD) incidence in California and Denmark, locations where large population studies have been done to determine the etiology of autism spectrum disorders [5]. The worldwide change point was determined to be 1988 [7]. An increase in incidence has been reported by the CDC to be as high as 800% [6] in the 10-year period between 1993 and 2003, as compared with 30% in all other disabilities. It is estimated that currently ASD affects 1 in 68 children in the USA, with an even more significant rate in boys of up to 1 in 42 [6].

## Etiological theories

The underlying etiology for ASD is largely unknown; however, there are many theories related to the pathogenesis of the disorder. The etiology is thought to involve genetic, neurobiological as well as environmental factors though the role of each individual factor is still being discovered. It has been previously shown that individual genetic mutations account for a relatively low percentage of the overall number of ASD cases*.* Single gene mutations can significantly increase the risk for developing ASD [7]. These include *FMR1* (fragile X syndrome), *MECP2* (Rett syndrome), *TSC1/TSC2* (tuberous sclerosis complex) as well as several others [8,9]. Reports vary regarding the impact of these single gene mutations toward the overall prevalence of ASD with some studies finding only 10% of diagnosed ASD patients having identifiable genetic mutations or syndromes [10] and others as high as 40% [11]. This wide difference between studies necessitated the search for other potential causes including several environmental factors that have been implicated as having associations with ASD development.

Connors *et al*. [12] described that the origin of ASD may involve multiple genes and can be influenced by environmental factors. They postulated that it is possible that perturbation of fetal development by environmental factors can lead to neurodevelopmental disorders without genetic cause. Environmental insults seem to have a significant role in the pathogenesis of ASD. There have been several studies researching environmental factors and their association with ASD development. One proposed association was thimerosal-containing vaccines based on data presented at the Institute of Medicine (IOM) Immunization Safety Review Committee in July 2001 by Blaxill. During this meeting, the IOM found insufficient evidence to accept or reject an association between thimerosal and neurodevelopmental disorders but found the hypothesis ‘biologically plausible’. Blaxill *et al*. subsequently published an article defending the ‘autism-mercury hypothesis’ [13]. Continued study, however, showed increased annual incidence rate of ASD cases in Sweden despite reduced levels of ethylmercury in vaccines [14]. Further studies have supported this evidence suggesting a lack of association for thimerosal-containing vaccines [15–17]. Childhood vaccination with the combined measles, mumps and rubella (MMR) vaccine [15,18,19] has also not been found to have an association with ASD development. The Immunization Safety Committee of the IOM determined in 2004 that the body of evidence favors rejection of a causal relationship between the MMR or thimerosal-containing vaccines and autism.

## Current thoughts on fetal role of ASD development

Though ASD was once considered a childhood disease, efforts for early detection led to the realization that it might originate at a much earlier stage, namely fetal life [12]. There are many environmental risk factors that have been postulated to be associated with the development of ASD including maternal hypertension, pre-eclampsia, diabetes mellitus, autoimmune disease and infection during pregnancy. At present, there are no randomized control trials showing causal relationships between individual maternal risk factors with the development of ASD. There are, however, several population-based studies with large numbers of participants evaluating these risk factors. These studies provide the groundwork to evaluate decisions on appropriate risk factor management for women that are pregnant or are considering pregnancy to lower the risk of ASD development.

We performed a review of the scientific literature to find any associations observed in fetal life that are linked to ASD development. To identify publications discussing associations between maternal environmental exposures and the development of ASD, a search of Pubmed and EMBASE databases through February 2015 was conducted to identify pertinent articles using the search terms ‘autism’, ‘pregnancy’, ‘ASD’, in all combinations with the terms ‘metabolic conditions’, ‘fever’, ‘autoimmune disorder’, ‘infection’ and ‘obesity’. Much of the data available were obtained through population-based studies. In many cases, data collection in these studies was not tailored only for ASD development. However, currently there are no experimental studies using animal models showing a causal effect between an environmental insult and the development of ASD; thus these population-based studies provide a platform for which to begin researching this important topic.

In this review, we would like to detail which associations have been determined through population-based studies to not be associated with the development of ASD.

## Epidemiological studies

Several large population-based studies have been done to determine the risk of ASD development. These studies have been done in several different population groups including American and European cohorts representing varied demographic backgrounds. According to Szklo, there are three primary advantages to the population-based study design [20]. The first advantage is the estimation of distributions and prevalence rates of variables within the population. Another advantage is that risk factor distributions measured at baseline can be compared with distributions in future cross-sectional samples so as to assess risk factor trends over time. The final advantage is that it allows for unbiased evaluations of relations, not only of confounders to exposures and outcomes, but also among any other variables of interest, even those which were not specified or identified in the original hypothesis.

The Childhood Autism Risks from Genetics and the Environment (CHARGE) study [21] was done in a California-based cohort. It is a population-based, case-control study of children from three groups: children with ASD, children with developmental delay (DD) without ASD and children with typical development (TD). Children with ASD and DD were recruited from lists provided by the California Department of Developmental Services; referred from the Medical Investigation of Neurodevelopmental Disorders (MIND) Institute, University of California, Davis, local physicians or regional centers that contracted with the California Department of Developmental Services; or self-referred after public outreach efforts. Population controls were selected randomly from California birth files with a male-to-female ratio of 4:1 and frequency matched for age and broad geographic regions within the study catchment areas. Inclusion criteria were age of 24–60 months, residence with at least one biological parent, English or Spanish spoken by at least one parent, birth in California and living within specified catchment areas in California. Children with severe visual, hearing or motor impairments that precluded standardized developmental assessment were excluded. Participants in this analysis were enrolled from 29 January 2003 through 7 April 2011.

The Nurses’ Health Study II (NHS II) is a prospective cohort of 116,608 female nurses aged 25–42 when recruited in 1989, who have been followed by biennial mailed questionnaires to assess the incidence of cancer and other chronic diseases. Completion and return of questionnaires sent by US mail constitutes implied consent. The 2005 questionnaire included an item asking women if they had a child diagnosed with autism, Asperger syndrome or ‘other autism spectrum disorder’. Only women who had given birth at least once before the end of 2003 and returned the 2005 questionnaire when the outcome was assessed were included; this provided a primary study population of 66,445 women. Among those women, 9477 had their first birth in 1989 or later and were included in the prospective subgroup sensitivity analysis, while 11,287 women had only one pregnancy and were included in the uniparous sensitivity analysis.

Several studies have been published in Sweden due to nationwide registries available for analysis. The Swedish population register system retains routinely collected health and sociodemographic data on the entire population of Sweden. The registers are cross-linked using a unique national registration number assigned to all Swedish residents at birth (or upon migration to Sweden) [22]. The ascertainment of ASD in the total Swedish population is based on data from national registers primarily covering inpatient admissions. One study's sample [23] comprised all individuals born in Sweden between 1984 and 2007, who were followed until 31 December 2011. All data were derived from linkages to national registers held by Statistics Sweden and the National Board of Health and Welfare. The National Patient Register contains data on all inpatient care in Sweden since 1973 and includes outpatient specialist care since 2001. ASD case status as of 31 December 2011, was defined as a recorded diagnosis of ICD-9 (299) or ICD-10 (F84) in the National Patient Register. A recent medical record review of autism in the National Patient Register following a CDC validation protocol confirmed the presence of DSM-IV autism in 83 of 88 persons (94.3%) [24]. In total, 2,385,678 persons were in the 1984–2007 birth cohorts. After exclusion of observations with missing covariate data, the final sample comprised 2,371,403 persons, with 24,414 identified ASD cases.

## Results

Currently, these studies present the only data available on these associations. Many factors were evaluated and many associations were discovered; however, follow-up studies will need to be done to ascertain whether true causative roles are present for the associations found.

Based on the CHARGE study, the following environmental factors failed to show a significant association with ASD development: Type 2 and gestational diabetes [25], hypertension [25], fever in first trimester [26], fever in the third trimester [26], fever treated with antipyretic medication [26], autoimmune disease [27], autoimmune disease of the thyroid [27], psoriasis [27] and rheumatoid arthritis [27].

Based on the NHS II maternal self-reported history of gestational diabetes failed to show a significant association with ASD development [28].

Based on data published from the Swedish nationwide registry the following environmental factors failed to show a significant association with ASD development: maternal diabetes [29], maternal hospitalization with any infection in the first trimester [23], maternal hospitalization with bacterial infection in the first trimester [23], maternal hospitalization with viral infection in the second trimester [23], an interpregnancy interval between 9 and 11 months compared with an interpregnancy interval greater than or equal to 36 months [30], and an interpregnancy interval between 12 and 23 months compared with an interpregnancy interval greater than or equal to 36 months [30].

## Discussion

Maternal hypertension, gestational diabetes and febrile illness during the first trimester are factors that are not associated with the development of autism spectrum disorder. The role of pre-eclampsia, and eclampsia might still play a role in causing inflammation within the cerebral cortex early in pregnancy, when the blood–brain barrier is still developing. This suggests that a deeper analysis of early fetal growth and maternal–fetal physiology may still unlock some answers as to the etiology of ASD. In 2013, the American College of Obstetricians and Gynecologists published a task force report on hypertension in pregnancy detailing the reconfigured diagnoses of hypertensive disorders of pregnancy along with revisions on management suggestions for patients with these disorders [31]. One of the referenced studies from this task force characterized the inflammatory response of pregnancy and pre-eclampsia including the time course of its evolution during pregnancy [32]. They found that cultured peripheral blood mononuclear cells (PBMCs) from pregnant women compared with those from nonpregnant women showed significantly enhanced secretion of IL-12, IL-18 and TNF-α in the first trimester and that there was significantly decreased IFN-γ in the first trimester. The response in pre-eclampsia was also altered in important ways, relative to matched normal pregnancy samples; IL-18 production was significantly increased and production of IFN-γ was no longer suppressed. Anti-angiogenic factors and disordered placentation leading to endothelial dysfunction has also been implicated in the clinical syndrome of preeclampsia [33]. An overexpression of sFlt-1, an endogenous anti-angiogenic protein that is made by the placenta and acts by neutralizing the proangiogenic proteins VEGF and PlGF, has been shown to produce proteinuria, hypertension and glomerular endotheliosis in pregnant rats, a phenotype consistent with pre-eclampsia. The relationship of these inflammatory and anti-angiogenic factors to ASD development requires further study.

There is also the possibility of a double-hit hypothesis where a genetic risk factor coupled with an environmental insult at a critical time period during fetal growth greatly increases the odds of ASD development. As detailed earlier, many genes have been postulated to be involved with the development of ASD, which can be potentiated by environmental insults. There appears to be at least one crucial time period between the first and second trimester where the associated risk of ASD development appears to be lower after this checkpoint has been reached. Further research needs to be undertaken to determine potential reasons for this heightened sensitivity prior to this checkpoint.

The strength of our analysis is the comprehensive literature search done within PubMED to find large studies that have provided exposure odds ratios connecting environmental risk factors to ASD. As this topic is currently largely unexplored there are no available controlled trials using animal models to connect the maternal–fetal physiology to ASD. Potential weaknesses of the studies include that the cohort within the Swedish population registry, while being the largest available to study potential ASD-risk factors, was not originally set up to study ASD and thus reflects post hoc analysis and that the NHS II study has the potential for recall bias due to its questionnaire format.

## Conclusion

ASD is a diagnosis with lifelong complications. Its prevalence is increasing at an alarming rate: an increase of greater than 100% in the last decade alone [6]. Children and adults with ASD have needs that challenge our systems of care. In addition to a great emotional toll that afflicts the family caring for an autistic child, the economic burden is immense on both parents as well as society [34,35].

Many of the associations that have been discussed in this paper appear to have increased odds ratio for the development of ASD however these values have not been shown to be significant by themselves, and the data preclude us to form deeper conclusions. Further research needs to be done in the future to further advance the knowledge about the etiology of this still mysterious disorder. The end goal of this research will be to improve screening of mothers identified as high-risk to have children with ASD for potential early evaluation and intervention that so far has been shown to be the only effective treatment available.

## Future perspective

In the next decade, we speculate that there will be increasing evidence to the fetal origins of ASD. As further light will be shed into the pathophysiology of this disorder as well as the maternal factors that are implicated in its genesis, fetal intervention as well as change in prenatal care, might lead to a decrease in its incidence. With further understanding of the factors that are truly associated versus not with ASD development, obstetric care can be modified to optimize the effect of these factors especially in key milestone stages of embryonic and fetal development. This can be through maternal dietary modification or management of underlying maternal conditions. In addition, current use of noninvasive prenatal screening using circulating free fetal DNA, and the availability of microarray genetic evaluation via the same technology, can lead to potential early diagnosis of fetuses at genetic risk for ASD. In the near future, we speculate this will be further studied, and at risk fetuses might benefit from modifications of risk factors, undergo gene therapy especially if they carry single genes implicated with ASD, or to start early intervention even earlier.

**Executive summary**

Autism spectrum disorder is a diagnosis with lifelong complications. Its prevalence is increasing at an alarming rate.
Currently ASD affects 1 in 68 children in the USA, with up to 1 in 42 boys.
ASD etiology is thought to involve genetic, neurobiological as well as environmental factors, however remains largely unknown.
A growing literature supports fetal origins of autism pathogenesis.
Large population-based studies have looked into various maternal conditions that can be associated with ASD.
These studies provide the groundwork to evaluate decisions on appropriate risk factor management for women that are pregnant or are considering pregnancy to lower the risk of ASD development.
Lowering anxiety for expecting mothers in knowing that some of these factors have not been proven as causative for autism in the offspring is of invaluable importance.
